# Dr. Edward Brown-Sequard’s Experimental and Clinical Researches, Epilepsy

**Published:** 1857-04

**Authors:** 


### *Dr. Edward Brown-Sequard's Experimental and Clinical Researches, applied to Physiology and Pathology.*

EPILEPSY—(Continued).

SECTION XI. I have been led to believe, by what occurs in animals after an injury to the spinal cord, and by some cases observed in man, that the existence of a particular spot capable of producing fits, when irritated, is not rare in epileptic patients. This spot may or may not be the starting-point of an aura epilepica.

In the interesting thesis of M. Bravais (*Rech. sur les Sympt. et le Traitement de l' Epilep.* Paris, 1827, p. 18), there is a case of a man who had fits when he touched himself, or was touched by other persons, on the region of the temporal bone of the right side.

Fernel, according to Esquirol (*loco cit.* p. 302), saw epilepsy produced each time pressure was made on the upper part of the head.

Rondelet (*Methode Curative des Maladies*, p. 137,) relates the case of a man who had a fit every time his ears were exposed to cold.

In a young man, in whom there was an aura epileptica starting from the left hypochondrium, a simple pressure on this region was sufficient to cause the fit (Tulpius, quoted by Portal, *loc. cit.* p. 180).

While I was lecturing on this subject in Boston, in November last (1856), Prof. E. H. Clarke told me that he had seen a fit of epilepsy produced by pressure upon one of the mammæ.

I have found that irritation of certain parts of the skin by galvanism caused fits in two epileptics. In one of them, it was the skin of the bend of the elbow, and in the other the skin of a portion of the neck and face. There was no sensation of an aura epileptica in these two cases.

Probably in many cases, without the feeling of an aura epileptica, and even without a feeling of pain arising from any part of the skin, the fits are caused by a peculiar and unfelt kind of irritation, originating from some part of the skin, or from the sensitive nerve of a muscle. Perhaps it will be possible to detect the existence of such parts of the external tegument, or of such nerves, by various means, of which we will speak hereafter. It is certainly impossible to admit that the sensations which exist when there is an aura epileptica are always the causes of the fits, as we know that sometimes they consist only in a feeling of cold, or a kind of tickling or formication, or a slight pain. Such sensations are certainly unable to produce fits, and therefore there must be some other kind of irritation, not felt, existing together with these sensations, starting from the same point, and producing the fit. Consequently, what is essential in the aura epilepica is not what is felt, but an unknown kind of irritation. This special irritation, we repeat, may exist alone, *i. e.* without any kind of sensation. It is the essence of an aura, without any feeling. A good illustration of this view may be found in some cases recorded by M. Pontier (see above, Sec. IX. Case VII.), J. Frank, and Henricus ab Heer. In the curious case we owe to M. Pontier, there was no pain arising from the feet, and, nevertheless, it is certain that an irritation sprang from them, as we find that the fits were prevented by the application of a ligature round the legs, and afterwards by the section of the saphena nerves. In the case mentioned by J. Frank (*Praxeos Medicæ Universæ Precepta*, Vol. I. Sec. III. p. 476), epilepsy had come after a disease of the testicle; the scrotum was much contracted during the fit, and, although there was no feeling of an aura, castration was performed, and the patient cured. It is evident that in this case the fits were due to an unfelt aura arising from the testicle. In the case by Henricus ab Heer (cited by Sennert, *Opera Omnia*, Vol. II. p. 489), a young girl had no feeling of an aura epileptica, but as she rubbed her big toes one against the other during the fit, applications of butter of antimony was made upon them, and the patient was cured. It seems that in this case, also, there was, as cause of the fits, an unfelt irrritation arising from the toes. It is well-known that worms in the bowels may cause epileptic fits, although they sometimes do not give pain or any other sensation. The irritation producing the fits is then unfelt, as in the preceding cases.

On one side, therefore, we find that an irritation coming from the skin or a mucous membrane may produce fits without being felt; whereas, on another side, when there is the feeling of an aura epileptica, the variety of the sensations, and their feebleness, often show that it is not they which cause the fit, so that we must admit that even then it is a peculiar, unfelt irritation which produces the attack. In my animals, as I have tried to prove in Sec. IV. it is not the pain caused by pinching the skin of a part of the face and neck which produces the fit, but a peculiar kind of irritation. Perhaps the special irritation which generates a fit gives sometimes a sensation quite special also, and which cannot be described. Many epileptics speak of a strange and inexplicable sensation. M. Delasiauve thinks he has been enabled to judge upon himself how a sympathetic fit is produced. He had a sore throat, with an engorgement of the cervical ganglions. The least pressure on these inflamed glands caused a sudden bewilderment (*eblouissement*). The experiment, repeated twenty times, always gave the same result. M. D. says, that if the pressure had been continued he would have fainted, and that there was quite a special sensation, progressing as quickly as a flash of lightning from the diseased spot to the head (*loco cit.* p. 33–34).

In the cases of epilepsy in which there is an unfelt irritation arising from the skin and producing the fits, is it because the irritation causes immediately a complete loss of consciousness, or because it has not the power of giving sensation, that it is not felt? I cannot answer this question positively. I can only say that it is probable that the two things exist.

If we take notice of these three sets of facts:—*First*, That there are cases of epilepsy in which an irritation arising from the skin, or from the neighboring parts, may cause fits without being felt; *Secondly*, That by pressure or galvanization we may produce in a part the kind of unfelt irritation which causes fits; *Thirdly*, That such a part being found, epilepsy may be cured by either the application of ligatures, the section of a nerve, or cauterizations, &c.:—It becomes evident that it is of the greatest importance to try to find out, in epileptics who have no aura epileptica, if there is not a part of the skin or of a muscle from which arises an unfelt irritation causing the fits. To ascertain the state of things in this respect, various means may be employed. If the fits are frequent, and if they come at regular times, it will be found, by placing tight ligatures around the limbs, whether the attacks are due to an irritation coming from these parts or not. Among other means of detecting the existence or absence of a peripheric irritating cause of the fits, I will point out particularly the following:—Pressure upon the various parts of the body; the application of localized and powerful galvanic currents; the application of ice, and of a wet and warm sponge, &c. If any part is the seat of a pain, even if this pain seems to have no relation with the fits, it will be necessary to ascertain whether pressure, galvanism, &c. applied upon this part, produce an attack. If it is in a limb that a pain exists, a ligature will decide the relation of the painful spot with the fits. In cases where there is a cramp in some of the muscles, or in one only, at the beginning of the fit, the inducement of a cramp by galvanism might decide if the attack is due to the irritation of the sensitive nerve of the contracted muscle, or if the cramp is nothing but a manifestation of the attack. If the initial cramp exists in a limb, an elongation of the contracted muscle, or a ligature, might lead to the solution of the question.*

The danger of producing a fit by the employment of some of the means that I have indicated as good to decide if there is an unfelt irritation arising from the skin, or from some muscle, and causing the fits, is not a reason to prevent our making use of these means; because the existence of a fit, particularly when we are prepared for it, is a small evil in comparison with the great benefit that may be derived from such a trial.

In my animals, nothing in the skin of the face and neck (except a slight congestion, which perhaps is the result of the pinching, and other modes of excitation that I employ,) indicates that this part has such a power as that which it alone possesses to cause fits when irritated. It results from this fact, that it would be quite wrong to decide, *a priora*, that an epileptic man, in whom the skin seems to be perfectly healthy, cannot have fits produced by an irritation of some parts of his skin. Even in such a case, therefore, it would be necessary to employ the various means I have indicated to decide the influence of the skin on the production of the fits.—*Boston Medical and Surgical Journal.*

* No one will imitate a surgeon (cited by Portal, *loco cit.* p. 135), who performed an amputation of one of the toes, because the movements of this toe were very violent during the fit!

